# Iliac Bone Tuberculosis Presenting as Left Thigh Swelling in an Indian Female Patient: A Rare Case

**DOI:** 10.7759/cureus.28297

**Published:** 2022-08-23

**Authors:** Sankalp Yadav

**Affiliations:** 1 Medicine, Shri Madan Lal Khurana Chest Clinic, New Delhi, IND

**Keywords:** tb, delayed reporting, coronavirus pandemic, skeletal tuberculosis, bone tuberculosis, covid-19, tuberculosis

## Abstract

Tuberculosis (TB) is a public health issue and is one of the main contributors to morbidity and mortality. The disease is very common in endemic areas. TB mainly occurs in the lungs; however, it can spread to other organs and the same is widely reported. Cancellous bone TB like that in the iliac bone is a rare finding even in endemic areas. The pandemic of coronavirus disease (COVID-19) has resulted in the delayed reporting of many cases. The present report is of a unique case of primary iliac bone TB in an Indian female who presented with painful swelling in her left thigh for three months. A detailed clinical work-up was done to establish the diagnosis. The delay was due to COVID-19 lockdowns and management was initiated after three months. This case highlights the importance of a high degree of suspicion to diagnose TB in rare extrapulmonary sites like the iliac bone.

## Introduction

Tuberculosis (TB) is a highly infectious disease caused by *Mycobacterium tuberculosis *and is a major health hazard [1]. The disease manifests as pulmonary or extrapulmonary TB [2]. Extrapulmonary TB is the result of the spread of bacteria from the lungs to other organs [2]. Extrapulmonary TB constitutes about 10-15% of all TB cases [3]. The pandemic of coronavirus disease 2019 (COVID-19) has impacted the care of other diseases [4]. There was a steep rise in the total notifications of TB cases after the third wave of COVID-19. Not only was there an increase in the number of cases reported but presentations of many rare or never seen before diseases were also recorded in the medical literature [4].

Primary iliac bone TB is extremely rare and poses a diagnostic challenge [5]. The situation becomes even more challenging when the patients report late. The delay in the present circumstances could be attributed to the COVID-19 pandemic [6]. Improper care received due to oversaturated healthcare system, local lockdowns, and fear among the general public of COVID-19 has led to delays in reporting of patients in the outpatient departments (OPD) [6]. The author presents a case of a 21-year-old Indian female who came after three months of delay post noticing the swelling in her left thigh. This delay led to the development of a large swelling, which was painful. However, prompt investigations and appropriate management were initiated. The main aim of this case report is to highlight the involvement of not so common sites of a common infectious disease in endemic areas and the successful management of the same during a pandemic.

## Case presentation

A 21-year-old Indian female belonging to a low socioeconomic background came to the OPD with chief complaints of pain and swelling in her left thigh for three months. She also reported an evening rise of fever with night sweats and loss of appetite for one month.

She was well three months back when she developed a painful swelling in her left thigh. To begin with, the swelling was small but it progressed to a very large size in the last three months. The pain had increased in the last 15 days and was aggravated on walking and relieved (slightly) when she rested. Fever was reported for one month, which was high grade, associated with night sweats, and subsided after taking an over-the-counter antipyretic (paracetamol). There were no chills, rigor, or cough. She did not notice any remarkable weight loss. There was no major medical or surgical history. She had no history of TB, COVID-19, seizure, or any trauma. Further, there was no history of similar complaints in the family or any of her contacts. And she had no history of recent COVID-19 vaccination.

**Figure 1:**
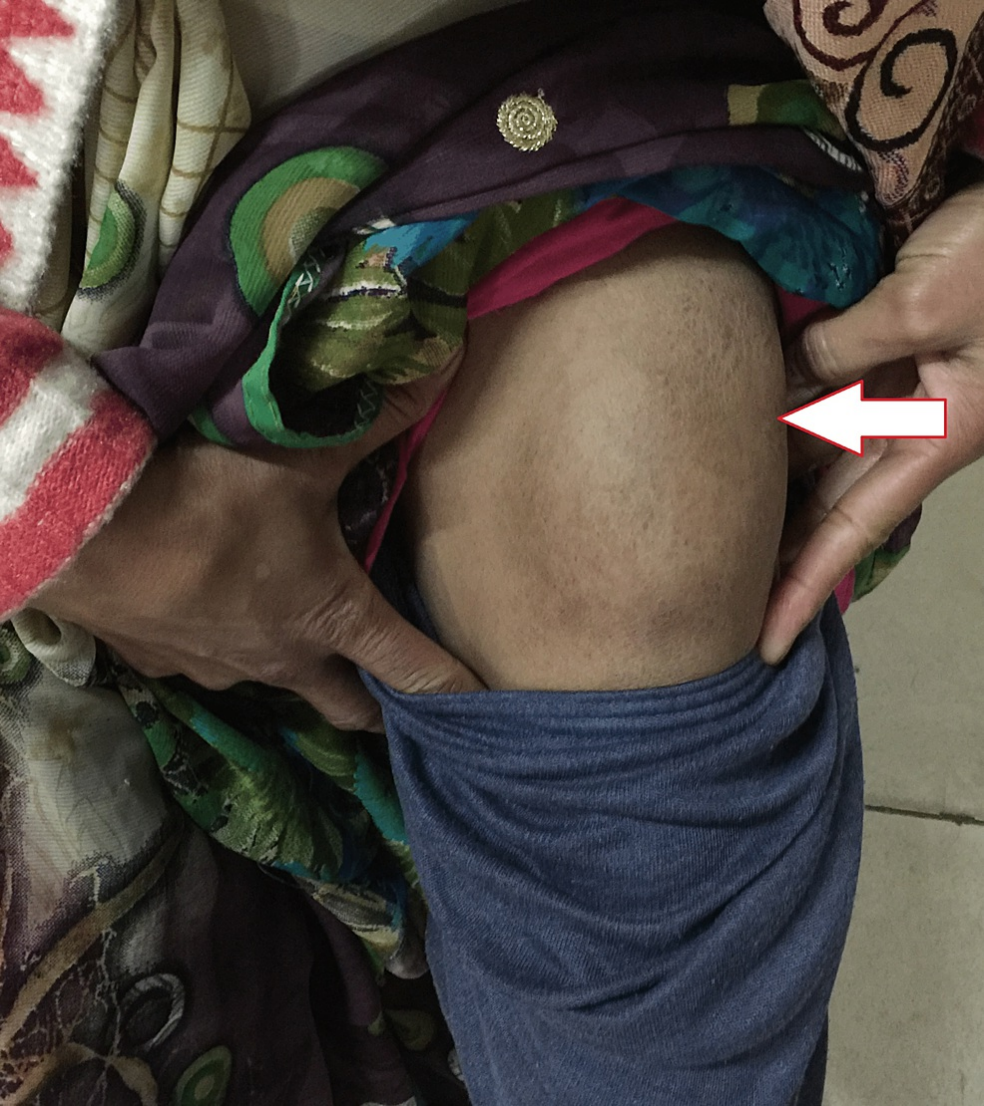
Large swelling in the proximal left thigh

General examination revealed an afebrile female with a pulse of 88/minute, blood pressure of 110/80 mmHg, respiratory rate of 19/minute, and peripheral capillary oxygen saturation (SpO2) of 98% on room air. Her systemic examination was unremarkable. Local examination revealed a large 20 X 10 cm swelling (Figure 1). It had a smooth surface, cystic in consistency, and tender to touch with well-defined margins. The swelling was mobile in the transverse direction. There was no raised temperature or any discharging sinus, and the swelling was non-fluctuant. The overlying skin was normal with no rashes. There was no clubbing, icterus, pallor, lymphadenopathy, cyanosis, edema, or koilonychia.

**Figure 2:**
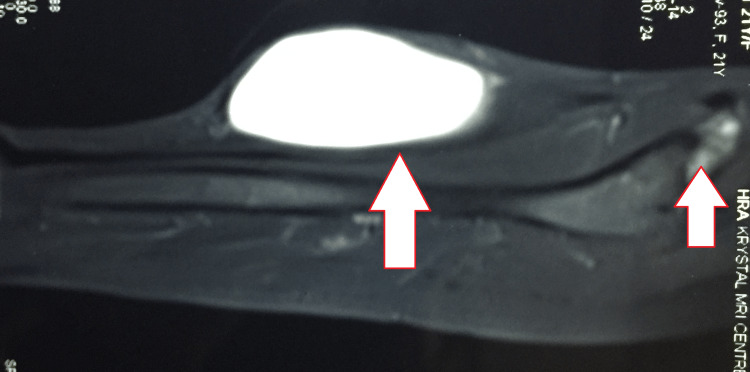
Magnetic resonance imaging of the pelvis and thigh

A provisional diagnosis of left thigh abscess was made and investigations were undertaken. The laboratory work-up was noteworthy for a positive tuberculin skin test and a raised erythrocyte sedimentation rate of 90 mm/hour. Her chest and spinal radiographs were normal. A radiograph of the pelvis showed a well-defined round lytic lesion in the body of the left iliac bone. A magnetic resonance imaging of the pelvis and thigh was suggestive of a well-defined Short-TI Inversion Recovery (STIR)/T2 hyperintense and T1 hypointense cystic intensity soft tissue lesion within the intermuscular plane of anterior thigh muscles of the proximal left thigh. The lesion had a long narrow neck extending superiorly above the left hip joint within the anteroinferior left iliac bone just over the anterior pillar of the acetabulum. There was a focal well-defined erosion in the anteroinferior left iliac bone through which the lesion was hanging down in the anterior thigh. Therefore, the lesion was pedunculated with origins from an anteroinferior part of the left iliac bone. The lesion had compressed and not invaded the muscles of the anterior left thigh. There was minimal soft tissue edema around the lesion and few septae were reported on the posterior part of this lesion (Figure 2). The cytology from fine-needle aspiration (FNAC) of the lesion was done and one sample was also sent for the cartridge-based nucleic acid amplification test (CBNAAT) and the culture and drug-susceptibility testing (DST). The FNAC was suggestive of inflammatory granulomatous diseases with a few multinucleated giant cells, epithelioid cells, and granular eosinophilic material, and the Ziehl-Neelsen staining for acid-fast bacilli positive. The reports of CBNAAT confirmed the same with *Mycobacterium tuberculosis* detected (low) with no resistance to rifampicin. The liquid culture system BACTEC (Becton, Dickinson and Company, Franklin Lakes, New Jersey, United States) identified culture was suggestive of *Mycobacterium tuberculosis* with no resistance to any drugs. The rest of the laboratory tests including HIV were unremarkable.

Finally, the patient was diagnosed with TB of the left iliac bone with a cold abscess in the left thigh. She was started on anti-TB treatment, as per the national guidelines of India, of four drugs i.e., rifampicin (450 mg), isoniazid (300 mg), pyrazinamide (1000 mg), and ethambutol (600 mg) for two months constituting the intensive phase and followed by rifampicin, ethambutol, and isoniazid for next four months in the continuation phase. At her request, she was transferred out to her village with advice for regular follow-ups in the Orthopedics OPD.

## Discussion

Extrapulmonary TB constitutes a substantial number of total TB cases. The disease spreads from a primary focus in the lungs to other organs. Skeletal TB is the second most common site for extrapulmonary TB after the lymph nodes [7]. The incidence of skeletal TB is less than 5% of all TB cases [5]. Further, skeletal TB is commonly reported in the spine and long bones and the same in cancellous bones is less common [7]. Furthermore, isolated iliac bone TB is extremely rare, and it accounts for less than 1% of all skeletal TB cases [5,7]. Iliac bone TB is usually reported in conjunction with sacroiliac TB and very few reports of isolated iliac bone TB are available in the literature [7]. Also, with few exceptions, iliac bone TB has been mainly reported in immunocompromised patients [7]. Isolated iliac bone TB in an immunocompetent female is a rare entity and it is especially rare in the absence of any previous history of TB.

The diagnosis of iliac bone TB is a formidable task and differential diagnosis should include Brodie's abscess, granulomatous lesions, chronic pyogenic osteomyelitis, and tumors like chondroblastoma, Kaposi sarcoma, osteoid osteoma or sarcoma, and non-Hodgkin lymphoma [8-10].

A case similar to this case was published by Trikha et al. [11]. However, the present case differs from their case in having a large swelling on the left thigh and by the nature of it being reported after a significant delay due to the COVID-19 pandemic. Another case by Ismail et al. reported iliac bone TB but the present case differs from their case in the location of the lesion, presence of the swelling in the thigh, gender, and delayed presentation [10]. A case reported by Elghoul et al. had similar presentations but this case differs from their case in presence of left thigh swelling, absence of a family history of TB, and medical management with no surgical intervention [5].

The author presented a case with large painful swelling, which was established as an outcome of left iliac bone TB. The patient reported late due to the third wave of the COVID-19 pandemic and its impact was evident on the delayed management of this case. The main aim of this case report is to highlight the adverse effects of a pandemic on the management of common diseases. The traffic restrictions, local and national lockdowns, and the widespread morbidity and mortality instilled fear among the general public due to COVID-19. This led to an unnecessary postponement of visits to health facilities thereby affecting the management of other infectious diseases.

## Conclusions

Skeletal TB such as iliac bone TB is a rare presentation. Diagnosis and prompt management involve a detailed history with judicious use of lab investigations and imaging modalities. There was a significant delay in the reporting of the case by the patient due to COVID-19 restrictions. In such cases, it poses a challenge for the management of the case and requires a high index of suspicion to rule out the rare extrapulmonary TB like that of the iliac bone.
